# Optimal control theory enables homonuclear decoupling without Bloch–Siegert shifts in NMR spectroscopy

**DOI:** 10.1038/s41467-018-05400-4

**Published:** 2018-08-01

**Authors:** Paul W. Coote, Scott A. Robson, Abhinav Dubey, Andras Boeszoermenyi, Mengxia Zhao, Gerhard Wagner, Haribabu Arthanari

**Affiliations:** 1 Department of Biological Chemistry and Molecular Pharmacology, Harvard Medical School, 240 Longwood Avenue, Boston, MA 02115 USA; 2 Dana-Farber Cancer Institute, 450 Brookline Ave, Boston, MA 02215 USA; 3 Department of Chemistry and Chemical Biology, Harvard University, Cambridge, MA USA

## Abstract

The Bloch–Siegert shift is a phenomenon in NMR spectroscopy and atomic physics in which the observed resonance frequency is changed by the presence of an off-resonance applied field. In NMR, it occurs especially in the context of homonuclear decoupling. Here we develop a practical method for homonuclear decoupling that avoids inducing Bloch–Siegert shifts. This approach enables accurate observation of the resonance frequencies of decoupled nuclear spins. We apply this method to increase the resolution of the HNCA experiment. We also observe a doubling in sensitivity for a 30 kDa protein. We demonstrate the use of band-selective C^*β*^ decoupling to produce amino acid-specific line shapes, which are valuable for assigning resonances to the protein sequence. Finally, we assign the backbone of a 30 kDa protein, Human Carbonic Anhydrase II, using only HNCA experiments acquired with band-selective decoupling schemes, and instrument time of one week.

## Abstract (web summary)

Bloch–Siegert shifts prevent the accurate observation of resonance frequencies in NMR experiments. Here the authors present a method for homonuclear decoupling that avoids inducing Bloch–Siegert shifts and improves the sensitivity and resolution of HNCA experiments.

## Introduction

In driven two-level quantum systems, the Bloch–Siegert shift is a perturbation of the observed resonance frequency caused by an off-resonance field^1–5^. The observed spin is subjected to its intrinsic Larmor precession as well as an oscillating field. After dynamical averaging of the oscillatory field, the resulting average Hamiltonian contains an apparent shift in the Larmor frequency, compared with its intrinsic value^6^. In nuclear magnetic resonance (NMR) spectroscopy, simultaneous application of radiofrequency (RF) pulses at a range of frequencies is common, especially for decoupling purposes. When the coupled spin is nearby in frequency to the observed spin, the decoupling pulse induces a Bloch–Siegert shift^7,8^. This prevents accurate observation of the resonance frequencies and causes severe phase distortions to the observed resonance peaks^9^. However, decoupling has important benefits: increased signal-to-noise ratio and improved resolution due to the collapse of multiplet line shapes. Decoupling is especially important in protein spectroscopy, because of the large number of resonances in limited spectral space.

The HNCA is a prototypical NMR triple-resonance experiment. It is necessary for sequential assignment of protein backbones^10–12^. For large proteins, its TROSY version^13^ is the most sensitive of the triple-resonance experiments that yield sequential connectivities^14^. For comparison, the sensitivity of the HNCACB experiment, typically used to resolve assignment ambiguities, is ~25% of the HNCA for high-molecular-weight proteins at high field^14^. In principle, the HNCA alone provides enough information for complete sequence-specific assignment. In practice, however, there is insufficient dispersion of *C*^*α*^ chemical shifts. This leads to degeneracies in peak position, which prevent unambiguous assignment.

Deuteration of the protein slows the *C*^*α*^ relaxation and increases the achievable resolution^15^. For large (≥ 30 kDa) deuterated proteins in high fields, resolution of 4–8 Hz in the *C*^*α*^ dimension is possible based on the relaxation rates^16^. However, with uniform sampling of the Nyquist grid, collecting sufficient indirect increments for this level of resolution would require instrument time on the order of months. However, high resolution is readily accessible, in feasible acquisition time (i.e., 2–3 days), using non-uniform sampling (NUS)^17–19^. In contrast, C^*β*^-encoding incurs relaxation losses due to longer delays for magnetization transfer and CO spins suffer from a large chemical shift anisotropy and relax quickly in high fields. Therefore, the achievable resolution is poor and the sensitivity is low for experiments that encode or transfer through *C*^*β*^ or CO. This suggests that the ability to assign challenging proteins using only the HNCA is of considerable interest.

The *C*^*α*^–*C*^*β*^ coupling splits the peaks into doublets in the *C*^*α*^ dimension. This *J*-splitting is undesirable because it halves the sensitivity and the larger footprint contributes to crowding and overlaps. There are several ways to decouple this splitting; however, each technique has its own difficulties. The constant time experiment^20,21^ lets the *C*^*α*^ evolve for a multiple of 1/*J*, so that the coupling modulation envelope refocuses. However, the *C*^*α*^ needs to be transverse for long times even to encode short indirect increments. To obtain high resolution, indirect time periods of up to 100 ms are needed and the peaks are almost completely lost to relaxation under a multiple constant time approach^16^. It is also possible to remove the splitting computationally, known as virtual decoupling^22^. The algorithm must be tuned to a specific coupling magnitude. However, any given protein can contain a range of couplings (33–42 Hz) and differences between the deconvolution setting and the actual coupling values lead to poor line shapes in the case of long indirect acquisition times^16^. A third scheme is to apply weak trains of decoupling pulses to the *C*^*β*^ regions. This produces singlets;^7^ however, the decoupling pulses generate Bloch–Siegert shifts. Correcting the peak list by approximately removing the effect (after acquisition) is possible by calibrating a best-fit model of the theoretical shift. However, this is cumbersome. Another significant problem with this technique is that, in practice, it does not produce the expected doubled peak height from collapsing a doublet into a singlet^7,22^, due to off-resonance effects from the decoupling field.

In this work, we use a single, optimized shaped pulse in the middle of the indirect encoding period to simultaneously refocus the *C*^*α*^–*C*^*β*^ and *C*^*α*^–*CO* couplings. The key insight is that decoupling can be done without causing any Bloch–Siegert shift in the *C*^*α*^ resonance frequency, by explicitly optimizing the pulse for the desired *C*^*α*^ behavior. As we require a different response from the *C*^*α*^ and nearby *C*^*β*^ resonances, we have used optimal control theory the design the shaped pulses^23–27^.

During the optimized decoupling pulses, the *C*^*α*^ spins precess according to their chemical shift frequencies, whereas the *C*^*β*^ and CO spins are inverted. The coupling refocuses during the second half of the encoding period. This approach is robust to different magnitudes of *J* couplings. We set up a cost/reward function that encourages *C*^*α*^ to follow its natural precession in the transverse plane while *C*^*β*^ and CO transition from + *I*_*z*_ to − *I*_*z*_. Pulsing on *C*^*β*^ necessarily perturbs the trajectory of the nearby *C*^*α*^ spins; however, Bloch–Siegert shifts can be avoided by incorporating a target final *C*^*α*^ state into the optimization. This approach avoids the severe phase distortions that arise if *C*^*β*^ is selectively inverted without controlling the behavior of *C*^*α*^. Phase distortions caused by the Bloch–Siegert shift are problematic even for inversion of the relatively isolated CO resonances, which is why the standard HNCA pulse program contains Bloch–Siegert compensation pulses^9^.

The shaped pulses we have designed here are named gradient optimized CO decoupling pulse (GOODCOP) and beta/alpha decoupling pulse (BADCOP). The latter has three variations targeted to specific *C*^*β*^ chemical shifts, BADCOP1, BADCOP2, and BADCOP3.

**Fig. 1:**
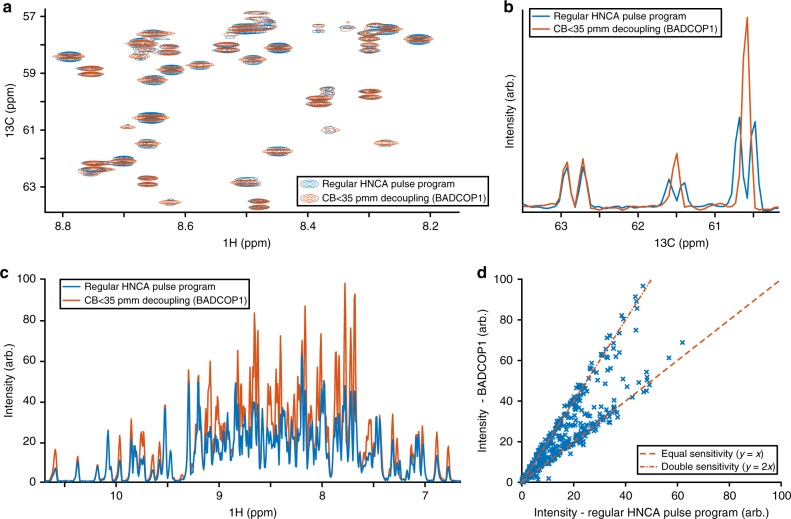
Sensitivity gain using BADCOP1. H_N_-C^*α*^ planes of HNCA were acquired on a 700 μM sample of HCAii, using the standard pulse program as well as with optimized decoupling of C^*β*^ below 35 p.p.m. and CO using BADCOP1. **a** Overlay of a section of the two spectra, showing that with BADCOP1 some peaks have collapsed into singlets in the C^*α*^ dimension, whereas some remain doublets, depending on *C*^*β*^ chemical shift. The full spectrum is in Supplementary Fig. 6. **b** 1D carbon trace through point H = 8.66 p.p.m. Decoupled peaks show the expected twofold gain in sensitivity, but not all peaks are decoupled. The decoupling pulse BADCOP1 has not caused any Bloch–Siegert shifts. **c** Skyline projection onto the 1 H axis (minus 1.2 arbitrary units to put the baseline at 0). The area under the curve is 48% larger for the version using BADCOP1. **d** Scatter plot to compare the two skyline projections. Two broad trends are visible: along the line *y* = *x*, the sensitivities are equal. Along the line *y* = 2*x*, the sensitivity is doubled by using BADCOP1. Partial decoupling, for C^*β*^ near the boundary of the inversion bandwidth, produces points between the two trend lines. Anything above the line *y* = *x* indicates a gain in sensitivity. All acquisition, processing, and display settings are the same for the two spectra

We have tested GOODCOP and BADCOP1–3 on two protein samples. The first is Protein G, B1 domain (GB1). The second is 30 kDa Human Carbonic Anhydrase II (HCAii) measured at 25 °C. By comparing the standard HNCA pulse program with our new method, we demonstrate that we induce no Bloch–Siegert shifts or phase distortions, and decoupled *C*^*α*^ peaks exhibit the expected doubling in sensitivity. Partially decoupled peaks, with *C*^*β*^ near the edge of the inversion bandwidth, also exhibit sensitivity gains. The sensitivity gain available by using BADCOP1 decoupling and the absence of any Bloch–Siegert shifts are demonstrated on HCAii in Fig. 1. Finally, we demonstrate that by using several different decoupling pulses (BADCOP1–3), we can extract sufficient residue-specific information for backbone assignment, without the need for additional triple-resonance experiments. We demonstrate 85% assignment of HCAii using only HNCA experiments, with GOODCOP and BADCOP1–3 decoupling pulses.

## Results

In this section we describe the newly developed pulse design methods, the experimental tests, and the assignment of HCAii using only the HNCA experiment.

### Design of GOODCOP for CO inversion with *C*^*α*^ encoding

GOODCOP is designed to have a different effect on *C*^*α*^ vs. CO spins. That is, all CO spins will be inverted, whereas the *C*^*α*^ spins will each precess at a rate proportional to their respective chemical shifts. The initial density matrix of any *C*^*α*^ spin, *ρ*(0), reaches a final state *ρ*(*T*) at the end of the pulse that has evolved under the unitary dynamics *U* = exp(− *iωaTI*_*z*_). Here, *T* is the pulse duration, *ω* is the chemical shift, *I*_*z*_ is the appropriate su(2) basis element for rotation about the *z*-axis (e.g., a Pauli matrix), and *a* is a constant that scales the rate of evolution. *T*′ = *aT* can be thought of as a contracted time, during which the spin evolves in the transverse plane at its intrinsic chemical shift frequency *ω*.

If *a* = 1 then there is no contraction; *T*′ = *T* and the *C*^*α*^ spins evolve at their natural chemical shift frequencies (as although there were no off-resonance pulse at all). However, provided that *a* is a constant we can ensure that there is no Bloch–Siegert shift by calculating the indirect increment accounting for the contracted time *T*′ = *aT*. In fact, other homonuclear decoupling methods also scale down the speed of the evolution in a similar way^28,29^ and a similar approach can be used to scale up *J*-splittings^30^. In our case, the scaling is only present during the relatively short pulse duration, not during the entire encoding period. Thus, the total (unscaled) time to encode a desired increment is only increased by a few microseconds because of the contracted time (which causes negligible relaxation compared with a maximum encoding time of up to 100 ms). As off-resonance pulsing on the coupled spin will interfere with the *C*^*α*^ dynamics during the pulse, setting *a* to a value < 1 allows the optimization some freedom for the *C*^*α*^ to deviate from their natural trajectory, as long as they end up at the desired final state at the end of the pulse. The desired behavior of the *C*^*α*^ is a universal rotation; we need the pulse to take any input state and produce the corresponding rotated output state.

In contrast, the required CO behavior is a point-to-point rotation. Specifically, if 165 < *ω* < 185 p.p.m., then we require only that longitudinal magnetization is inverted (i.e., *ρ*(0) = *I*_*z*_ is inverted to *ρ*(*T*) = − *I*_*z*_). Regardless of the initial spin state of the CO (e.g., transverse or longitudinal), this point-to-point specification is necessary and sufficient for decoupling.

Based on numerical experimentation and on hardware requirements, we chose a pulse duration of 150 μs, a maximum RF amplitude of 15 kHz, and set *a* = 0.9. This means that the *C*^*α*^ will acquire phase of *ωT*′ = *ω*(0.9*T*) during the pulse, i.e., *T*′ = 135 μs of evolution time at the intrinsic chemical shift frequency *ω*. Therefore, for a desired indirect evolution period of *t*_1_, we should let the spins evolve for *d*_0_ = 0.5(*t*_1_ − *aT*), then run the shaped pulse, then evolve a further *d*_0_. This takes *t*_1_ + 15 μs in real time, but only produces *t*_1_ worth of chemical shift encoding. There is 15 μs of overhead. The coupling changes sign in the middle of *t*_1_, when we run the pulse, and therefore refocuses exactly at the end of the encoding period. The minimum possible evolution time with the GOODCOP pulse is 135 μs, which occurs when *d*_0_ = 0. A conditional statement (“if statement”) skips the pulse for shorter indirect increments. Pulse sequence diagrams for this scheme, for regular HNCA as well as TROSY-HNCA, are given in Supplementary Fig. 1 and Supplementary Fig. 2.

The pulse shape optimization was conducted by sampling a set of 100 chemical shifts uniformly spaced in the *C*^*α*^ range *ω* ∈ [35,75] p.p.m. We alternate their initial states between *I*_*x*_ and *I*_*y*_, and set their desired final states to *ρ*(*T*) = *Uρ*(0)*U*^†^ where *U* = exp(− *iωaTI*_*z*_), *a* = 0.9, and *T* = 150 μs. By linearity, we can expect appropriate input/output behavior for any initial state in the transverse plane. We can also expect that any longitudinal magnetization will be preserved for these chemical shifts, since the net rotation is constrained to the *z*-axis. We further sampled 30 chemical shifts from the CO range *ω* ∈ [165, 185] p.p.m. and set their initial states to *I*_*z*_ and desired final states to − *I*_*z*_. We randomized the initial pulse shape and ran the GRAPE algorithm. We used our new implementation of GRAPE, set up in a toggling frame, to ensure that optimization converged quickly^31^. This speed helped a great deal when experimenting numerically with pulse duration and other parameters.

Experimentally, the pulse decouples the CO without producing any Bloch–Siegert shifts in the *C*^*α*^ resonance frequency (Supplementary Fig. 3). In contrast, using an off-resonance decoupling pulse such as WURST or hyperbolic secant leads to clear Bloch–Siegert shifts (Supplementary Fig. 4). Spectra recorded with GOODCOP on HCAii and GB1 are essentially identical to spectra acquired with a standard HNCA pulse program (although we note that our approach saves 576 μs of *C*^*α*^ relaxation compared with the standard method, which uses an intricate system of extra pulses and delays to address the Bloch–Siegert shift). In particular, the sensitivity is the same and the peaks appear in the same (unshifted) locations, without any phase distortions. Next, we expand the method to include *C*^*β*^ inversion, which is far more useful.

### BADCOP for CO and *C*^*β*^ inversion during *C*^*α*^ encoding

In this subsection, we explain the BADCOP1 design method. Specifically, we add the requirement that *C*^*β*^ is inverted during the pulse, so that the *C*^*α*^–*C*^*β*^ coupling can be refocused.

We chose a pulse duration of 1 ms. In general, longer pulse durations could produce more selective decoupling. Note that this 1 ms of pulsing occurs during the *C*^*α*^ encoding delay, and therefore it does not contribute additional time or relaxation losses to the experiment. Instead, the overhead is (1 − *a*)*T* (i.e., < 100 μs) due to the slightly slowed encoding of *C*^*α*^ during the pulse. This slowed encoding is uniform across the *C*^*α*^ bandwidth and is accounted for by adjusting the indirect evolution time as described above; therefore, it creates no Bloch–Siegert shift.

**Fig. 2:**
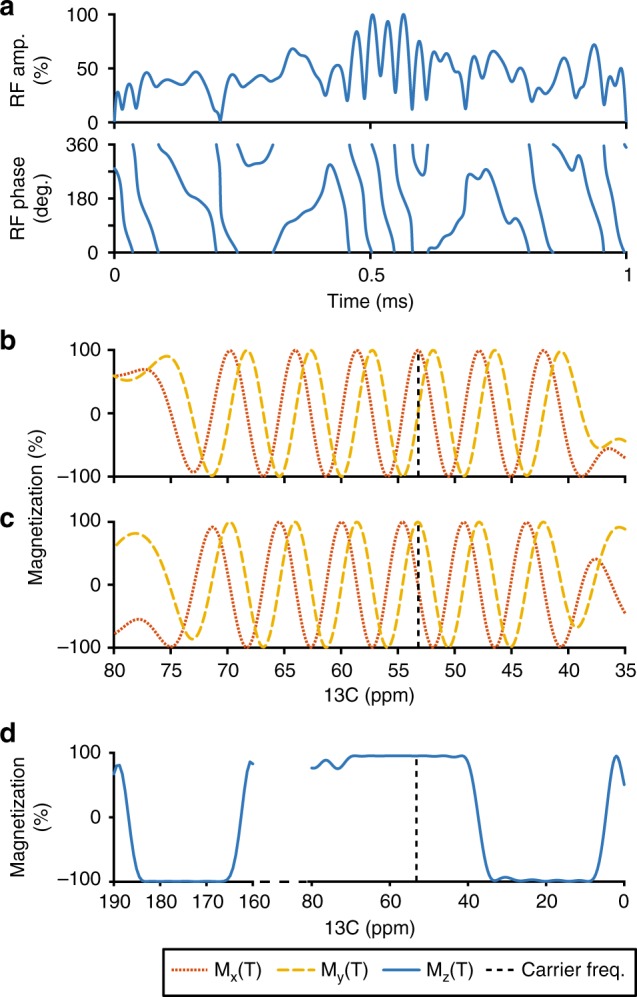
RF profile and simulation of BADCOP1. **a** The shaped pulse “BADCOP1” RF amplitude and phase profiles. Note that there are no abrupt changes in amplitude or phase. On an 800 Mhz spectrometer the maximum power is 5.94 kHz (100% amplitude) and the duration is 1 ms. For other field strengths, these values can be scaled appropriately to maintain the same bandwidths and flip angles. **b** Simulation assuming an initial state of *ρ*(0) = *I*_*x*_. We see sinusoidal chemical shift encoding in the transverse plane for the *C*^*α*^ region. This behavior was explicitly built into the optimization. Outside of the *C*^*α*^ region off-resonance effects take over and the encoding is lost. **c** Simulation assuming an initial state of *ρ*(0) = *I*_*y*_ also shows encoding. By linearity, the pulse is producing a universal rotation about the *z*-axis for the *C*^*α*^ region, with rotation angle proportional to chemical shift frequency. **d** Simulation assuming an initial state of *ρ*(0) = *I*_*z*_. The longitudinal magnetization is inverted for all CO and for *C*^*β*^ below about 35 p.p.m. This leads to selective decoupling of resonances in these two regions. The pulse can be run in the middle of an arbitrary-duration indirect encoding period to refocus resonances, without inducing any Bloch–Siegert shifts

We sampled 100 chemical shifts *ω* ∈ [40,72] p.p.m., alternated their initial states between *I*_*x*_ and *I*_*y*_, and set their desired final states to *ρ*(*T*) = *Uρ*(0)*U*^†^ where *U* = exp(− *iaωTI*_*z*_), *a* = 0.91, and *T* = 1 ms. Further chemical shifts were sampled, 30 from *ω* ∈ [165, 185] p.p.m. and 60 from *ω* ∈ [5,37] p.p.m., and for these the optimization was set up to require that longitudinal magnetization is inverted. The value of *a* = 0.91 was determined by numerical exploration, but other values *a* close to *a* = 1 also work well. The resulting optimized pulse is BADCOP1, which is depicted in Fig. 2 along with spin-dynamics simulations. The trajectories of individual spins are given in Supplementary Fig. 5. In particular, the *C*^*α*^ spins take a highly irregular trajectory during the pulse, but at the end of the pulse they end up in the same state as if they had evolved without any decoupling pulse for the contracted time *T*′ = 910 μs. At the same time, the *C*^*β*^ and CO spins take different trajectories, but end up inverted at the end of the pulse. The spin trajectories during the pulse are all highly intricate, in order to simultaneously achieve the various desired spin-state transitions for C^*α*^, C^*β*^, and CO with high fidelity.

### Band-selective *C*^*β*^ decoupling with CO inversion

Two additional BADCOP pulses were designed to selectively invert subsets of the *C*^*β*^ bandwidth. Specifically, we follow the general setup of the previous subsection, but vary the chemical shift range that is inverted. We also specifically optimize the pulses to not invert (i.e., to preserve) longitudinal magnetization for C^*β*^ outside of the desired selective decoupling bandwidth. The idea is to encode information about the C^*β*^ from the C^*α*^ line shapes in the HNCA, instead of from the HNCACB experiment. As the additional transfer time in the HNCACB (compared with the HNCA) results in large relaxation losses, selective decoupling of certain *C*^*β*^ is a viable alternative to transferring magnetization to and from *C*^*β*^, especially for high-molecular-weight systems.

Table 1 summarizes the pulses we have tested, including GOODCOP and the various versions of BADCOP. In particular, the pulse BADCOP3 inverts chemical shifts *ω* ∈ [10,45] p.p.m. This includes *C*^*β*^ for most amino acids. The exception is the two downfield shifted *C*^*β*^ of serine and threonine. However, for this pulse the desired *C*^*α*^ behavior (precession in the transverse plane) is achieved only above ~47 p.p.m., which excludes the glycine *C*^*α*^. As a result, glycine *C*^*α*^ are perturbed (i.e., they have reduced amplitude and/or are poorly phased) with BADCOP3.

**Table 1:** *C*^*α*^ decoupling pulses for the HNCA experiment

Pulse	Behavior	Duration *T* (ms)	Maximum RF amp. (kHz)	Contracted time *a*
[Regular pulse program]	We use the Bruker pulse program “hncagp3d.” There is no *C*^*β*^ decoupling, so all *C*^*α*^ are split into doublets (except Glycine).	NA	NA	NA
GOODCOP	CO is inverted while *C*^*α*^ evolves. There is no *C*^*β*^ decoupling, so all *C*^*α*^ are split into doublets (except Glycine). 576 μs shorter than “hncagp3d”.	0.15	15.00	0.9
BADCOP1	*C*^*α*^ evolves, CO and C*β* < 35 p.p.m. invert. Many peaks collapse into singlets.	1	5.94	0.91
BADCOP2	*C*^*α*^ evolves, CO and C*β* ≈ 28–35 p.p.m. invert, other *C*^*β*^ are not inverted.	1	4.87	0.91
BADCOP3	*C*^*α*^ evolves, CO and C*β* < 43 p.p.m. invert, some glycine *C*^*α*^ are not observed. Only serine and threonine residues show any *C*^*α*^ splittings.	1	7.22	0.91

Depending on which pulse is used, different splitting patterns are observed for different residues. Acquiring multiple HNCA with different decoupling schemes can narrow the choice of assignments considerably. In all cases, the pulse generates *aT* worth of *C*^*α*^ chemical shift evolution, so the pulse must occur between two encoding delays of duration *d*_1_ = 0.5(*t*_1_ − *aT*), where *t*_1_ is the desired indirect increment. Durations and RF amplitudes are quoted for an 800 MHz spectrometer, however these can be scaled appropriately for other field strengths to maintain the same bandwidths and flip angles

**Fig. 3:**
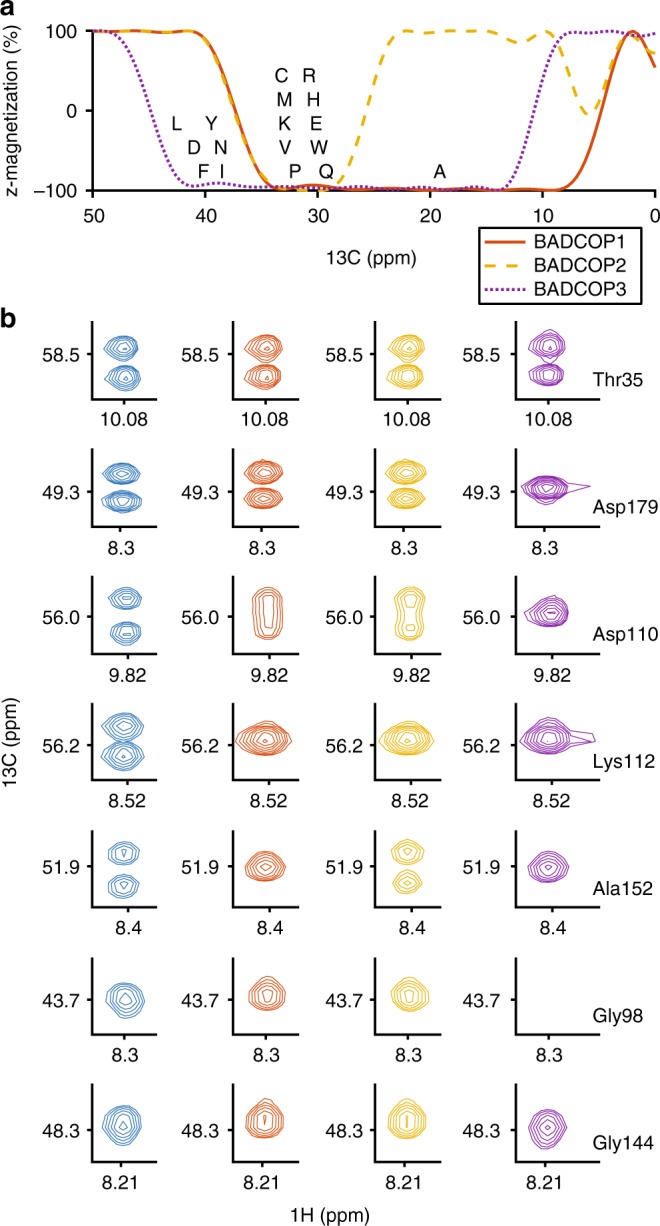
Experimental observation of amino acid-specific line shapes using BADCOP1–3. **a** Simulated C^*β*^ inversion profiles with the three decoupling pulses BADCOP1–3. The initial state is *ρ*(0) = *I*_*z*_, and the final longitudinal magnetization is depicted. The mean C^*β*^ chemical shift from the BMRB is indicated for the various amino acids. **b** Peaks selected from four 2D HNCA planes of HCAii, acquired with the standard pulse program (left column) and with the three different optimized decoupling pulses (columns 2–4; BADCOP1–3). The same peak is represented in each row. The peaks are either singlets or doublets depending on C^*β*^ chemical shift. Therefore, the patterns code for the C^*β*^ and indirectly for the amino acid type. The selectivity is not perfect in all cases (due to secondary shifts moving the *C*^*β*^). Note that there are no Bloch–Siegert shifts; all peaks appear at the correct *C*^*α*^ position (i.e., the same as the standard sequence in the leftmost column). Of particular interest is the loss of upfield glycine *C*^*α*^ (row 6) using BADCOP3 and the slightly different line shape patterns between the two aspartic acid *C*^*α*^, indicating different *C*^*β*^ chemical shifts (rows 2 and 3). The alanine pattern is unique (row 5). All acquisition, processing, and display settings are the same for the four spectra

**Fig. 4:**
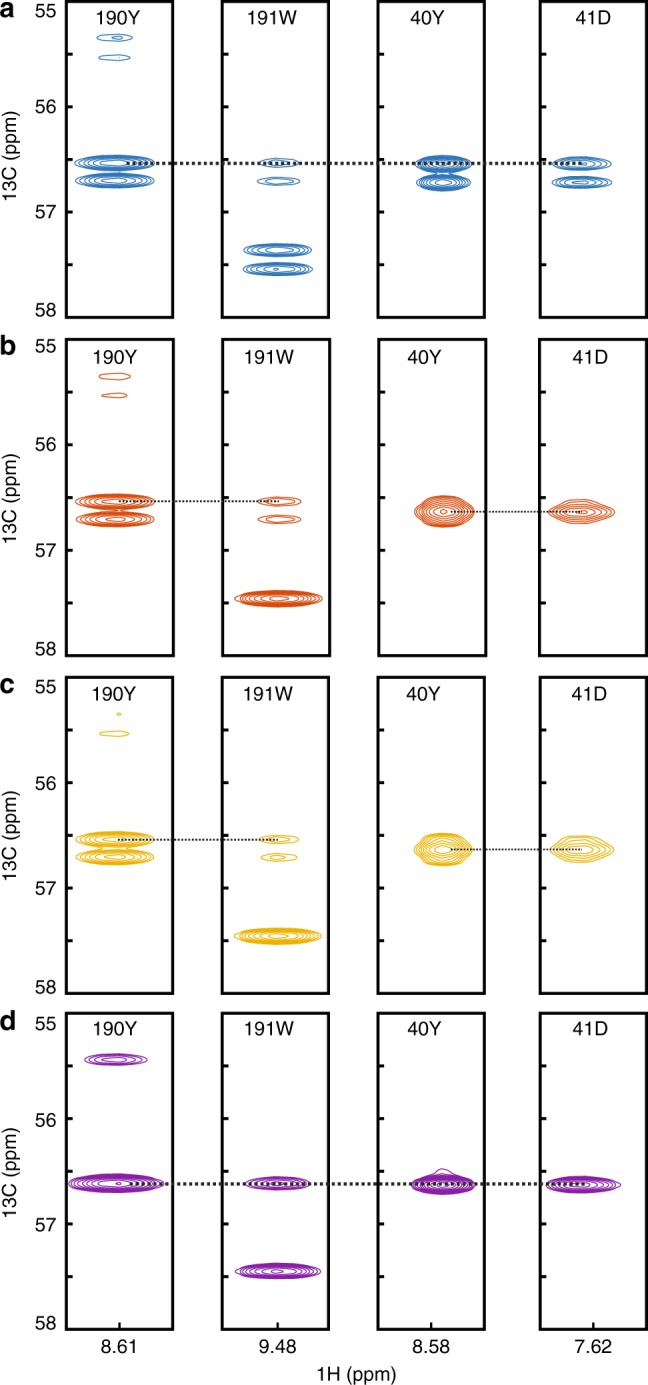
Using BADCOP1–3 line shapes and splitting patterns for sequential matching. In the 3D HNCA of HCAii, tyrosines 190Y and 40Y are overlapped in the *C*^*α*^ dimension, as are the sequential peaks in their respective *i* + 1 residues. This produces an ambiguity in the sequential assignment, which could be resolved using an HNCACB experiment. **a** The four HNCA strips, recorded using GOODCOP. All peaks are doublets and the chemical shifts are the same, generating an ambiguity in the sequential assignment. **b** Using BADCOP1, two of the peaks are partially decoupled and become broad singlets. The other two remain doublets. This resolves the ambiguity in the sequential matching process and implies that 190Y and 40Y have different *C*^*β*^ chemical shifts. **c** Using BADCOP2, the same decoupling pattern appears in this case. In some cases (depending on *C*^*β*^ shift), different line shapes are observed with BADCOP1 vs. BADCOP2. **d** With BADCOP3, all the peaks appear as singlets with doubled height compared with the doublets. The sequential peak for 190Y at 55.4 p.p.m. is more clearly visible above the noise. All acquisition, processing, and display settings are the same for the four spectra

**Fig. 5:**
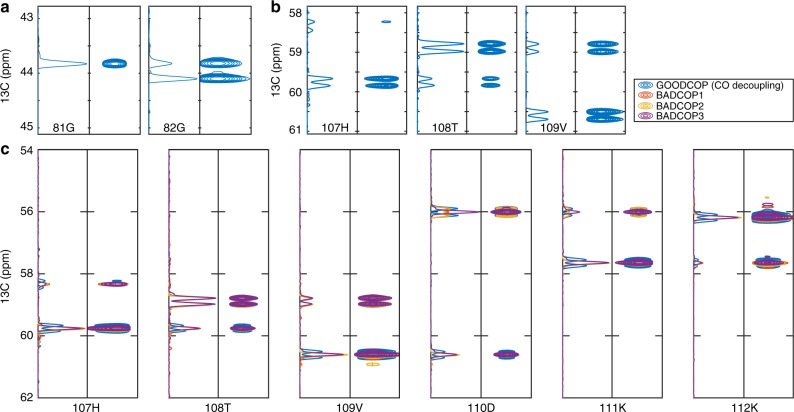
Using BADCOP1–3 line shapes and splitting patterns for sequential assignment. **a** Strips from a 3D HNCA experiment of HCAii. Glycines appear as singlets and have *C*^*α*^ chemical shifts around 45 p.p.m. The strip identified as “82 G” has an internal and a sequential glycine peak, and the sequential is matched to the strip “81 G”. Two neighboring glycines only occur once in the primary sequence, so these strips can be unambiguously assigned. **b** In contrast, most peaks appear as doublets because of the C*α*–C*β* coupling. These can be be sequentially matched, but assignment onto the primary sequence is difficult without further data or long chains of sequential correlations. **c** Using three BADCOP decoupling pulses gives residue-specific information. The three leftmost strips (the same strips as in **b**) show a particular sequence of different splitting patterns under the various decoupling pulses, as described in Fig. 3a. This pattern is only consistent with one location on the primary sequence of HCAii and so these can be unambiguously assigned. Further sequential matches show splitting patterns consistent with the primary sequence and the expected splitting patterns based on Fig. 3a. This gives many opportunities to make or confirm assignments. All acquisition and processing settings are the same for the four spectra, while strip contour levels are set individually for visual clarity. An expanded version of this figure is included as Supplementary Fig. 8

The benefit of this suite of decoupling pulses is that by acquiring multiple spectra, and examining which resonances are doublets or singlets in each, we can identify the approximate *C*^*β*^ frequency and narrow the choice of sequential candidates during the assignment procedure. Figure 3 shows examples of line shapes that we observe using the various BADCOP pulses and how these line shapes encode *C*^*β*^ chemical shift frequencies. It is worth mentioning that splitting patterns and line shapes are always identical in the internal peak and its sequential match. That is, if a certain peak is a singlet under one decoupling pulse and doublet for another decoupling pulse, then it can only be matched to a peak that follows the same pattern. In particular, this is true right at the boundary of the inversion bandwidth where we can see partially decoupled peaks with distinctive line shapes (i.e., with some narrowing and sensitivity gain). In general, true matches have a high correlation coefficient (between the internal and sequential line shape) irrespective of decoupling sequence^32,33^. In cases of chemical shift degeneracy, correct matches must correlate well for all decoupling pulses. If there is any exception, then they have different *C*^*β*^ frequencies and are therefore not a correct match. We show examples of how matching across multiple spectra, acquired with different decoupling pulses BADCOP1–3, resolves ambiguities in Fig. 4 and Supplementary Fig. 6. Specifically, some candidate sequential matches can be excluded, because their appearance using BADCOP1 is not the same as the appearance of the internal peak, even though the *C*^*α*^ resonance frequency is identical. This is analogous to resolving ambiguities using the HNCACB. Examples of how the additional information can be used to assign sequential matches onto the primary sequence are given in Fig. 5. Generating different line shapes in order to dramatically reduce the number of possible assignments can also be approached biochemically, using selective *C*^*β*^ isotope labeling^16^.

In the next section, we show that these homonuclear decoupling pulses perform well in experiments on simple (GB1) and challenging (HCAii) protein samples. In particular, we show that the *C*^*β*^-specific information is sufficient for assignment.

### Experimental validation on GB1

We have applied the optimized homonuclear decoupling pulses in HNCA experiments, and compared the results with the standard implementation. The decoupled *C*^*α*^ peaks do indeed become singlets and that the sensitivity gain is substantial (i.e., double).

GB1 is well known as a simple NMR model system. We recorded H_*N*_-C^*α*^ planes using the HNCA pulse program, in order to observe the resulting line shapes and verify the basic operation of the optimized decoupling pulses. We tested GOODCOP by collecting two-dimensional (2D) H_N_ − C^*α*^ planes with no decoupling at all vs. with GOODCOP. The results are as expected; the 55 Hz coupling to the CO is removed. Moreover, no Bloch–Siegert shifts are present (Supplementary Fig. 3). Next, *C*^*β*^ decoupling was tested by applying BADCOP. We acquired 2D H_N_ − C^*α*^ planes with various selective C^*β*^ decoupling schemes. In Supplementary Fig. 6, we show how the resulting line shape depends on the *C*^*β*^ chemical shift. This can be used to resolve ambiguous sequential correlations and narrow the choice of possible assignments.

### Backbone assignment of HCAii using BADCOP

The protein HCAii has a molecular weight of 30 kDa with a correlation time of 18 ns at 25 °C, which represent reasonably challenging conditions on which to test the GOODCOP and BADCOP decoupling pulses.

Initially, we acquired 2D H_N_ − C^*α*^ of the HNCA. We collected four 2D planes: one plane using the regular HNCA pulse program and the other three planes using *C*^*α*^ − *C*^*β*^ decoupling pulses BADCOP1–3. To measure the overall sensitivity we took skyline projections onto the ^1^*H* axis. Figure 1c shows these projections for the regular pulse program as well as for one of our decoupling pulses, BADCOP1. It is clear that a majority of peaks display higher intensity in the presence of decoupling using BADCOP1. To roughly quantify the overall sensitivity gains, we can calculate the area under the skyline projections. Compared with the regular pulse program, we see an overall enhancement of 48%. A more nuanced measure of the sensitivity improvement is to compare the skylines point-by-point, as in Fig. 1d (and also Supplementary Fig. 7E–G). In these scatter plots, two broad trends are readily apparent. Along the line *y* = *x* the two skylines are approximately equal, indicating no sensitivity gain. Along the line *y* = 2*x*, BADCOP1 decoupling has collapsed doublets into singlets for a twofold improvement. Similar analyses were done for the other BADCOP pulses and all these spectra showed substantial improvements in sensitivity (Supplementary Fig. 7). Depending on the decoupling pulse, we see different densities of points along the two trend lines (equal and doubled sensitivity).

In particular, BADCOP3 decouples *C*^*β*^ up to about 43 p.p.m. This interferes with the glycine *C*^*α*^ encoding. Therefore, we see many instances of degraded performance (points below the line *y* = *x*), corresponding to affected glycines (Supplementary Fig. 7F). We also see that most other peaks have doubled intensity; the line *y* = 2*x* is densely populated. If we restrict the skyline projection to *C*^*α*^ > 47 p.p.m. then the glycines are excluded from the projection (Supplementary Fig. 7G). Most of the peaks from the other residues fall on the double-sensitivity line, *y* = 2*x*.

We also analyzed four three-dimensional (3D) HNCA spectra of HCAii. We obtained amino acid-specific information by examining the splitting patterns under different decoupling pulses and this allowed us to assign the backbone using only a set of HNCA experiments acquired in less than 1 week of total instrument time. We emphasize that our 3D spectra are recorded using NUS, in order to access high resolution in the *C*^*α*^ dimension.

The observed resonances were classified into possible amino acids using the coding of line shapes and splitting patterns (Fig. 3) and sequentially matched (Fig. 4). Short chains of sequential correlations could then be unambiguously located on the primary sequence (Fig. 5). Longer chains of correlations provide additional confidence in the assignments (Supplementary Fig. 8 and Supplementary Fig. 9). Proceeding in this manner, 85% of the sequence was assigned. Practically speaking, almost all the observed amide resonances were successfully assigned. However, only around 85% of the expected number of systems were observed in the spectrum. The missing peaks are largely from the core of the protein, suggesting that there has been incomplete back-exchange of deuterons during sample preparation. Our spectra are missing these peaks altogether, preventing complete assignment.

### Comparison with existing decoupling pulses

Standard selective decoupling pulses shift the C^*α*^ resonances away from their intrinsic positions. However, we have designed our pulses to avoid such Bloch–Siegert shifts. A second drawback of selective dynamical decoupling using established shaped pulses is that the glycine *C*^*α*^ peaks are perturbed due to their proximity to the decoupling region^7^. This introduces strong cycling sideband artifacts, i.e., spurious satellite peaks that take magnitude away from the true peaks.

We have collected 2D H_N_ − C^*α*^ planes of HCAii using the methods of Matsou et al.^7^, with the same acquisition and processing parameters that were used to test the GOODCOP and BADCOP pulses. We observe the strong decoupling sidebands on glycine, and also observe smaller sidebands for other (non-glycine) peaks (Supplementary Fig. 4 and Supplementary Fig. 10). These satellite peaks reduces the height of their respective main peaks and make the spectrum more difficult to interpret. Moreover, the magnitudes of the Bloch–Siegert shift are different depending on which sort of selective pulses is used (e.g., WURST or hyperbolic secant) and exactly where they are centered. This makes it difficult to compare various spectra recorded with different pulse sequences, magnetization transfer pathways, and field strengths. Furthermore, accurate ^13^C chemical shift data are required for bond angle determination in structure calculations^34^, so decoupling pulses that influence observed resonance frequencies are to be avoided.

In addition to the unwanted Bloch–Siegert shifts, we also compared the sensitivity of selectively dynamically decoupled spectra (using WURST and hyperbolic secant) to our approach using GOODCOP and BADCOP. For CO decoupling only, the sensitivities are almost identical for the standard pulse program, selective decoupling, and GOODCOP. However, selective decoupling using either WURST or hyperbolic secant (30 p.p.m. decoupling band, centered at 170 p.p.m.) introduces Bloch–Siegert shifts. These spectra are shown in Supplementary Fig. 4. When decoupling *C*^*β*^ in addition to CO using WURST or hyperbolic secant, we do not observe the expected doubling of sensitivity. Unlike BADCOP, selective decoupling with WURST or hyperbolic secant suffers from sidebands and off-resonance effects, which diminish the heights of the real peaks and therefore lower sensitivity. We compared the empirical sensitivity for various attempts at selective decoupling and it is clear that spectra recorded with BADCOP have significantly higher overall sensitivity (Supplementary Fig. 11).

## Discussion

We have presented practical methods for homonuclear decoupling pulse design that completely avoid Bloch–Siegert shifts. The pulses were designed using the methods of optimal control, aided by rapid convergence techniques. The same pulses are applicable for other experiments that encode *C*^*α*^. For example, GOODCOP can be used to decouple *C*^*α*^ from CO in a ^13^C-HSQC and the series of BADCOPs can be used to remove the coupling from *C*^*β*^ in addition to CO. The use of these decoupling pulses in ^13^C-HSQC will especially find use in residual dipolar coupling measurements.

More generally, the pulse design methods we have developed are readily applicable to other homonuclear decoupling applications, as long as there is some frequency separation between the observed and decoupled spins. For example, one can remove *C*^*γ*^ and *C*^*β*^ couplings and record high-resolution ^13^C-HSQC for methyl resonances for amino acids such as alanine, isoleucine, and valine, where the methyl resonances are separated from the coupled side-chain resonance. This pulse design can also be used to remove ^13^C − ^13^C coupling of DNA/RNA bases and polysaccharides. In addition, similarly designed pulses can be used to remove ^19^F − ^19^F couplings without any Bloch–Siegert shift. Similar to GOODCOP and BADCOP, any such decoupling pulse can be transferred to any field strength by appropriate scaling of the duration and RF power level.

The decoupling pulses that we have designed perform very well for collapsing the *C*^*α*^ doublets in the HNCA. The Bloch–Siegert shift was removed by explicitly optimizing for *C*^*α*^ to continue precessing while *C*^*β*^ and CO invert. By targeting the inversion to a few specific *C*^*β*^ regions, we generated sufficient amino acid-specific information for assignment using only HNCA. Experimental tests show the expected doubling of sensitivity for decoupled spins, in contrast to previous efforts to dynamically decouple *C*^*β*^ from *C*^*α*^. Therefore, the new decoupling shaped pulses are highly suitable for NMR spectroscopy.

## Methods

### Sample preparation

First, we used a sample of 1 mM GB1. The sample is fully ^13^C, ^15^N labeled, and perdeuterated, and created following established protocols^35^.

Second, we used a ^13^C, ^15^N-labeled sample of the protein HCAii at a concentration of 700 μM. The correlation time is 18 ns at 298 K. We expressed and purified HCAii following established protocols^36,37^. Briefly, we transformed BL21(DE3)pLysS *Escherichia coli* cells (EMD Millipore) with a pACA plasmid containing the gene for HCAII (a kind gift from Carol Fierke and coworkers at the University of Michigan) and grew the transformed cells to an OD_600_ of 0.4–0.8 in M9 minimal medium (6 g/L Na_2_HPO_4_, 3 g/L KH_2_PO_4_, 0.5 g/L NaCl, 0.25 g/L MgSO_4_, 14 mg/L CaCl_2_, 100 M ZnSO_4_, 2 g/L d-^13^C-glucose (Cambridge Isotopes), 2 g/L ^15^NH_4_Cl (Cambridge Isotopes), and 100 mg/L ampicillin) in D_2_O. The medium was further supplemented with the trace elements (50 mg/L EDTA, 8 mg/L FeCl_3_, 0.1 mg/L CuCl_2_, 0.1 mg/L CoCl_2_, 0.1 mg/H_3_BO_3_, and 0.02 MnCl_2_) and the vitamins Biotin (0.5 mg/L, Sigma) and Thiamin (0.5 mg/L, Sigma). Protein expression was induced with 0.25 mM isopropyl *β*-d-1-hiogalactopyranoside and 450 mM ZnSO_4_ were added to ensure full Zn-ion occupancy in the HCAii active site. Protein expression was allowed to continue for 20–24 h after induction, at 25 °C.

We then collected the cells by centrifugation at 5000 r.p.m. for 15 min and purified ^2^H-, ^13^C-, and ^15^N-labeled HCAii following a previously described procedure^38^, with small modifications. Briefly, we re-suspended the cells into BPER protein extraction buffer (Thermo Scientific), containing 1 mM MgSO_4_, 1 mM *N*-p-tosyl-l-arginine methyl ester, 3 mM tris(2-carboxyethyl) phosphine, 2.5 mM ZnSO_4_, and 1 M phenylmethanesulfonyl fluoride. We then lysed the cells with a tip sonicator (VWR Scientific) at 70% amplitude, added 0.125 μg/mL lysozyme (Sigma) and 10 U/mL DNase I (Life Technology) to the lysed solution and incubated it in an incubator shaker for 2 h. HCAii was subsequently purified from cell lysate with (i) two rounds of precipitation with ammonium sulfate at room temperature (60% and 90% v/v with a solution of saturated ammonium sulfate, respectively), (ii) dialysis into Tris-SO_4_ buffer (50 mM in H2O, pH = 8.0) at 4 °C, (iii) anion exchange chromatography with a Q Sepharose Fast Flow resin (GE Healthcare), (iv) size-exclusion chromatography with a Superdex 75 resin (GE Healthcare), and (v) dialysis into NMR buffer (10 mM Na2HPO_4_/NaH_2_PO_4_ in H_2_O, pH = 7.6). Finally, we incubated HCAii overnight at 40 °C to exchange amide-bound deuterons to protons and added 5% D_2_O for NMR measurements. All measurements were recorded on a 700 μM sample.

### NMR experiments

NMR data collection of GB1 samples was performed on a Bruker 800 MHz instrument equipped with a cryogenically cooled TXO probe. The 2D H_N_ − C^*α*^ plane of a TROSY-HNCA pulse sequence in Supplementary Fig. 2 was used. Sweep widths for the ^1^H and ^13^C dimension were 12820 and 6036 Hz, respectively. Data collection on the HCAii sample was performed on a Bruker 750 MHz instrument equipped with a cryogenically cooled (TCI) probe. The TROSY-HNCA pulse sequence in Supplementary Fig. 2 was used. Sweep widths for the ^1^H, ^15^N, and ^13^C dimension were 12,019, 2735, and 4901 Hz, respectively. The indirect dimensions were sampled non-uniformly, selecting 458 out of a matrix of 40 × 300 (^15^N ×^13^C) complex points (4% sampling). The sampling schedule was selected based on the Poisson gap sine weighted protocol.

### Code availability

The pulse sequences (Supplementary Fig. 1 and Supplementary Fig. 2), GOODCOP/BADCOP shaped pulse files, and parameter set (all for Bruker Spectrometers) can be obtained from http://artlab.dana-farber.org/downloads. This webpage also has instructions for implementing the pulses at any magnetic field strenth.

### Data availability

Other data supporting the findings of this manuscript are available from the corresponding author upon reasonable request.

## Electronic supplementary material

Supplementary Information
