# Supplementary material for: Optimal control theory enables homonuclear decoupling without Bloch–Siegert shifts in NMR spectroscopy
Source: Nat Commun. 2018 Aug 1;9:3014. doi: 10.1038/s41467-018-05400-4 (PMC6070575; doi:10.1038/s41467-018-05400-4)
Supplement: Supplementary file 1 — Supplementary Information [file 41467_2018_5400_MOESM1_ESM.pdf]

OPTIMAL CONTROL THEORY ENABLES HOMONUCLEAR DECOUPLING WITHOUT BLOCH-SIEGERT SHIFTS IN NMR SPECTROSCOPY

Coote et al

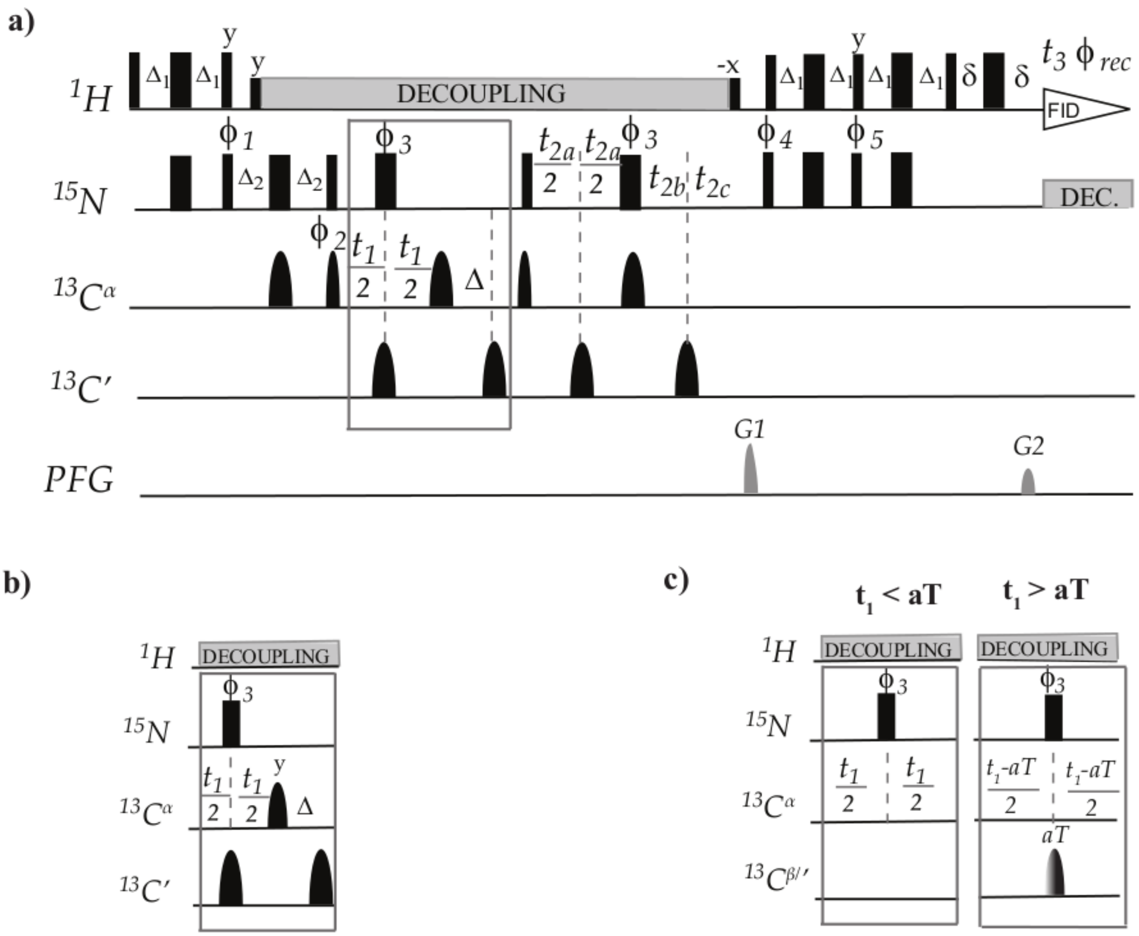

Supplementary Fig. 1. **3D HNCA pulse program.** (**a**) The regular HNCA pulse program. The boxed region includes indirect C^α evolution for an increment time period of t_1, during which a weak π pulse on CO refocuses the C^α-CO coupling. This soft pulse induces a Bloch-Siegert shift on the C^α resonances. An additional soft π pulse on C^α, and delays and a repeat of the soft π pulse on CO, correct for that Bloch-Siegert shift, which costs 576 μs of additional relaxation. (**b**) The section that we modify. Using GOODCOP or BADCOP, this section is replaced by (**c**), with GOODCOP/BADCOP depicted on the CO axis. The system of soft pulses and delays to remove the Bloch-Siegert shift is not needed (and if BADCOP1-3 is being used then C^β are being decoupling in addition to CO). In (**c**), we let the C^α evolve for $t_1 - aT$ in order to record the t_1 indirect increment, where T is the GOODCOP/BADCOP pulse duration, and a is the time-contraction factor as explained in the text. For very short increments ($t_1 < aT$), we skip the pulse.

Narrow and broad rectangular black bars indicate non-selective $\pi/2$ and π pulses, respectively. Black narrow and wide semi-elliptical shapes on the carbon channel represent $\pi/2$ and π Gaussian cascade pulses (selective for the frequencies of C^α/C^β or CO carbon nuclei), respectively. All pulses are applied along the x-axis unless otherwise indicated. The delays are $\Delta_1 = 1/4J_{HN} = 2.3$ ms; $\Delta_2 = 1/4J_{NCa} = 12$ ms. The phase cycle employed was $\psi_1 = (x, x, x, x, x, x, x, x, -x, -x, -x, -x, -x, -x, -x, -x)$; $\psi_2 = (x, -x)$; $\psi_3 = (x, x, x, x, -x, -x, -x, -x)$; $\psi_4 = (x, x, -x, -x)$; $\psi_5 = (-y, -y, y, y)$; $\psi_{rec} = (x, -x, -x, x, x, -x, -x, x, x, -x, -x, x, x, -x)$. Phase sensitive spectra in the indirect C^α dimension (t_1) are obtained by incrementing the phases ψ_2 in a States-TPPI manner and ^{15}N dimension (t_2) using Echo-Antiecho. The two smoothed-square shaped pulsed field gradients were applied along the z-axis for 1.0 ms with maximum intensities of G1 (80%) and G2 (8.1%). Deuterium decoupling are achieved by using WALTZ-65 and GARP.

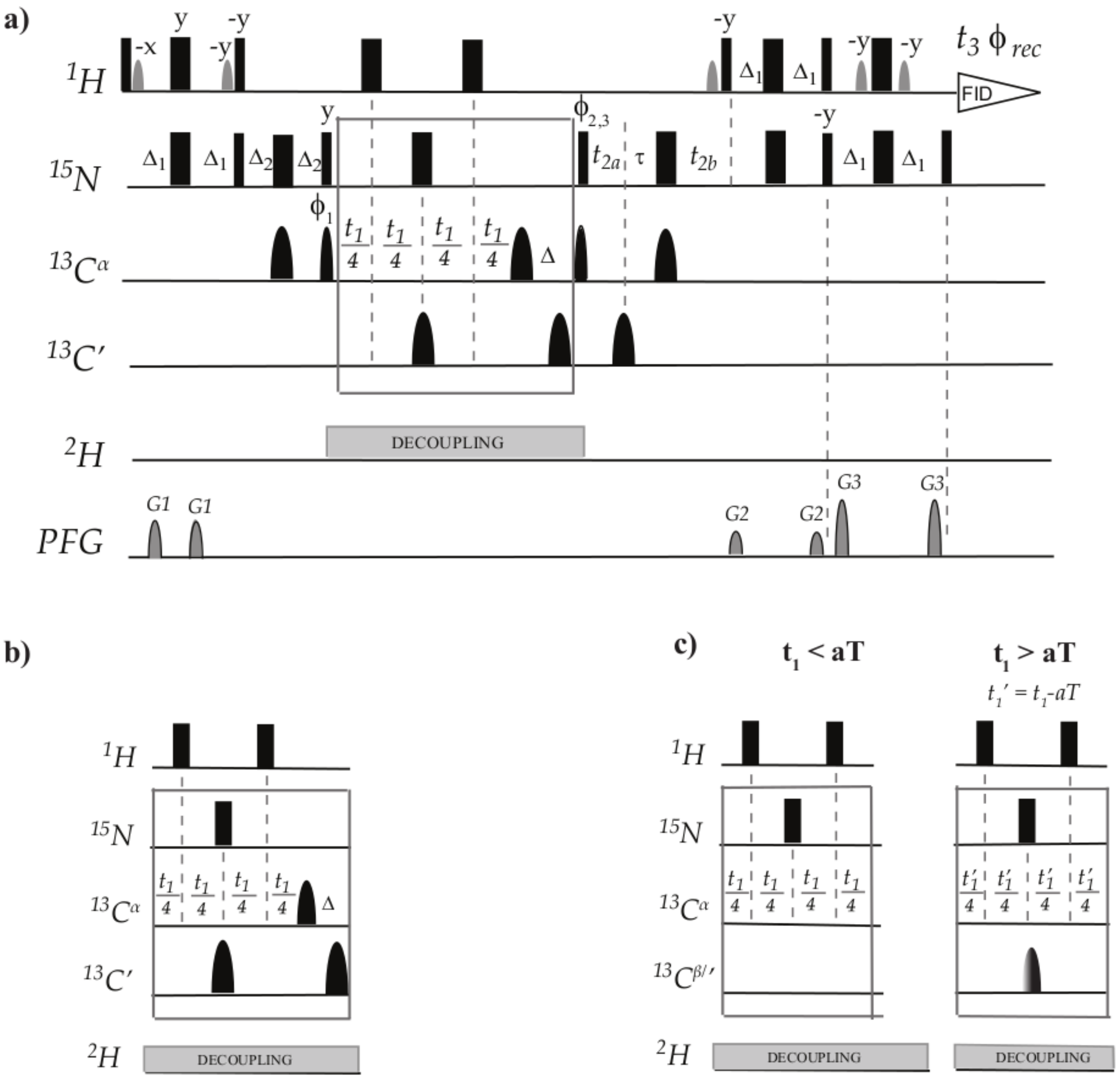

Supplementary Fig. 2. **3D Trosy-HNCA pulse program.** (**a-c**) The same as Supplementary Fig. 1, but with trosy selection. Additional minor differences compared to Supplementary Fig. 1 are needed for TROSY selection. Specifically, the phase cycles were $\psi_1 = (x, x, x, x, -x, -x, -x, -x)$; $\psi_2 = (y, -y, x, -x)$; $\psi_{rec} = (y, -y, -x, x, -y, y, x, -x)$. The three smoothed-square shaped pulsed field gradients had maximum intensities of G1 (30%), G2 (18%), and G3 (44%). Deuterium decoupling is achieved by using WALTZ-16.

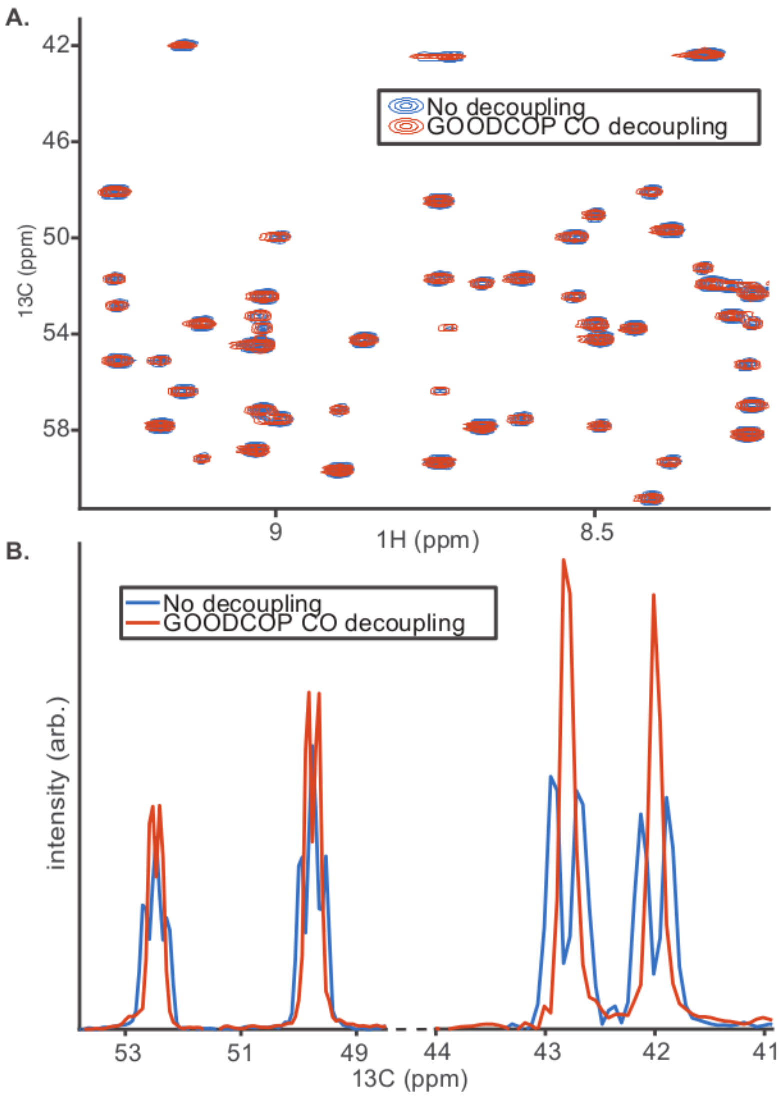

Supplementary Fig. 3. **CO decoupling using GOODCOP.** (**A**) 2D H_N-C^α planes from HNCA of GB1 using no homonuclear decoupling, versus using GOODCOP for CO inversion. We observe narrowing in the C^α dimension as the 55 Hz coupling to CO is removed. (**B**) 1D traces through the planes, showing the carbon line shapes. The triplets collapse into doublets (the C^α-C^β coupling is still present). Although the peak volume is unchanged by adding decoupling, we do not observe much sensitivity gain, due to the relative sizes of the splittings and linewidths. For glycine peaks around 42 ppm, there is no C^β, and removing the coupling to CO provides the expected twofold sensitivity gain. There are no Bloch-Siegert shifts. All acquisition, processing, and display settings are the same for the two spectra.

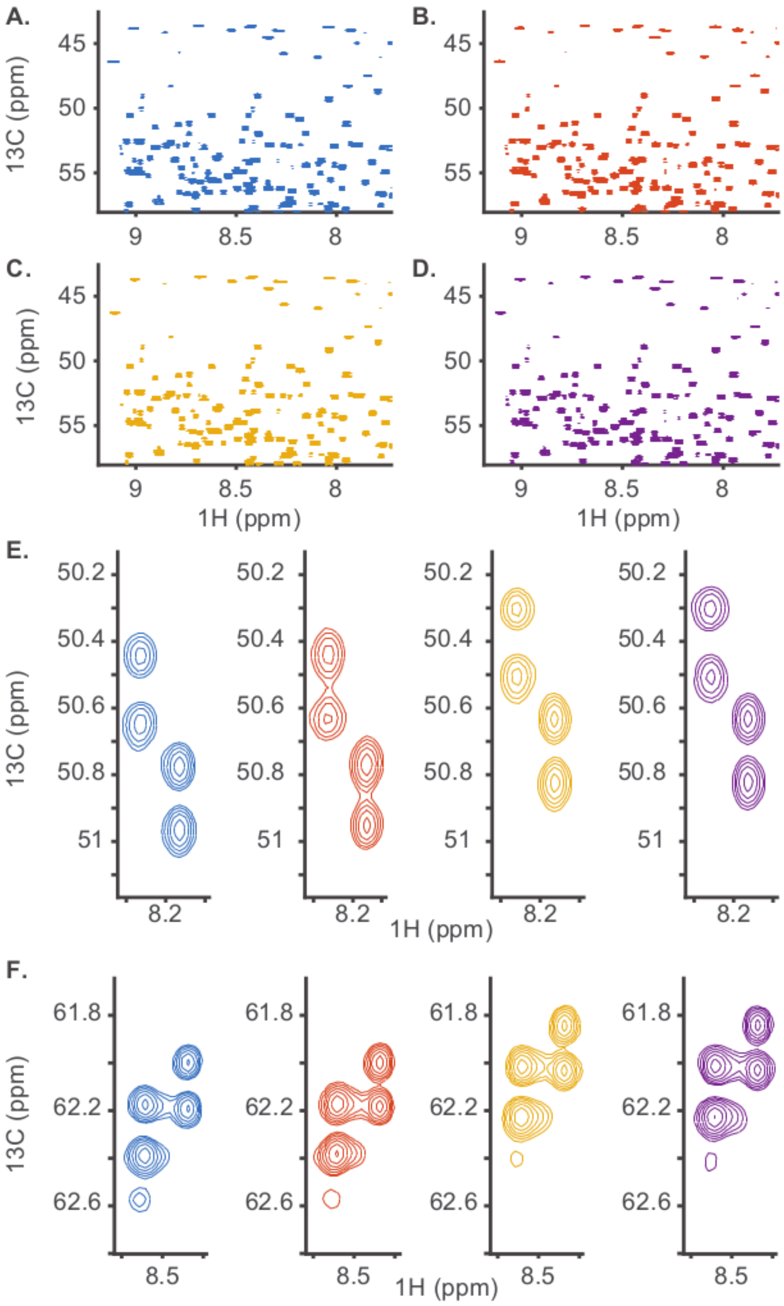

Supplementary Fig. 4. **CO decoupling with GOODCOP produces no Bloch-Siegert shifts.** GOODCOP was tested alongside two common decoupling pulses on the protein HCAii. (**A**) Using the regular Bruker pulse program 'hncagp3d'; (**B**) using GOODCOP to selectively invert CO while simultaneously controlling C^{α} encoding; (**C**) using hyperbolic secant decoupling of CO; (**D**) using WURST decoupling of CO. All four approaches produce good spectra, with nearly identical sensitivity. However, only the standard HNCA pulse program and GOODCOP avoid Bloch-Siegert shifts in the C^{α} dimension. (**E,F**) Two examples of specific peaks taken from each of the four spectra. Hyperbolic secant and WURST have shifted all peaks upwards (away from CO), in accordance with Bloch-Siegert shift theory. In contrast, the spectra produced using GOODCOP (second column) is effectively identical to the standard pulse program (first column). The GOODCOP approach saves 572 μs compared the the regular program, however we do not observe that this has had any effect on peak-height in this case.

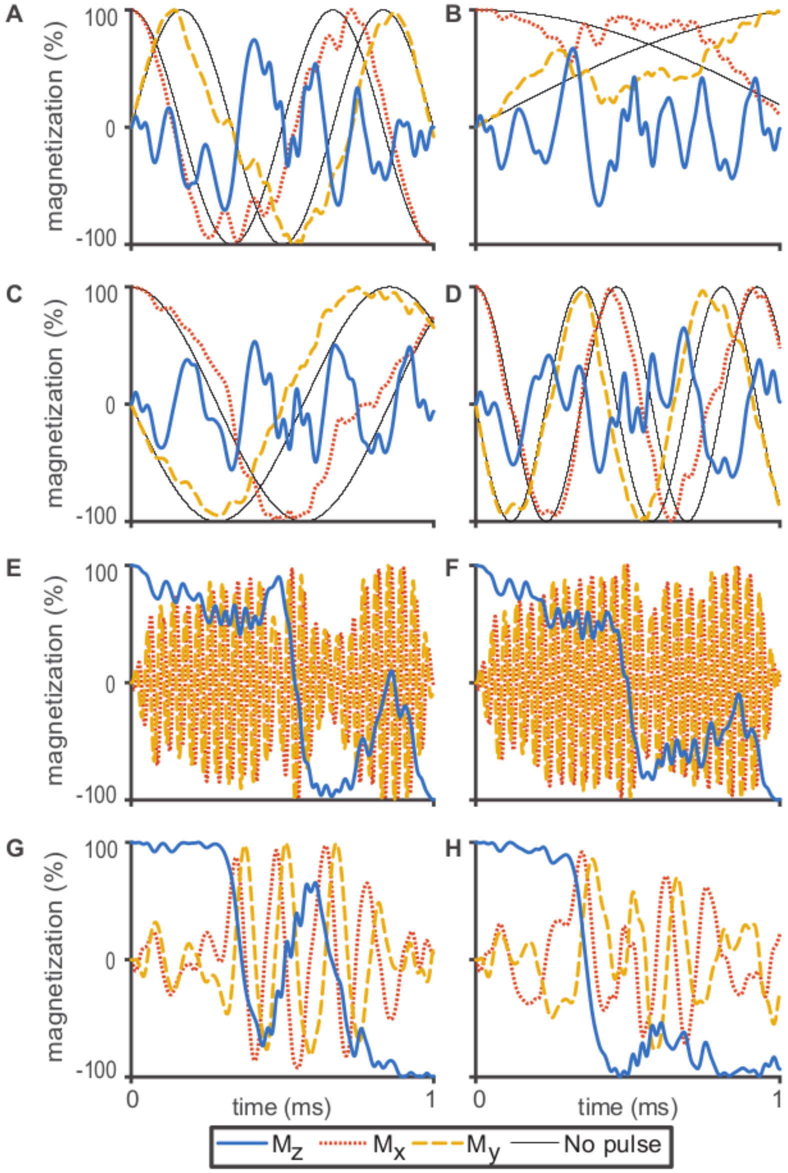

Supplementary Fig. 5. **BADCOP1 spin-dynamics shows highly intricate trajectories.** Simulated magnetization trajectories under the shaped pulse 'BADCOP1' for relevant initial conditions and chemical shifts. (**A-D**) $\rho(0) = I_x$, and chemical shift ω is sampled from the C^α bandwidth; $\omega \in \{45, 52, 58, 65\}$ ppm respectively. Faint gray lines show the trajectory for a scaled chemical shift of 0.91ω—equivalent to ω-encoding for the contracted duration $T' = 0.91T = 910$ μs without any decoupling pulse. Although spins take highly irregular routes during the pulse, at final time $T = 1$ ms they end up in the same final state as if they had evolved without any pulse for 910 μs. (**E-H**) CO and C^β longitudinal magnetization inverts during the pulse, which leads to decoupled spectra. $\rho(0) = I_z$ and $\omega \in \{173, 178, 20, 30\}$ ppm respectively. All trajectories are expressed in a rotating frame at the C^α carrier frequency of 53.2 ppm (hence the rapid precession visible for CO resonances in **E** and **F**).

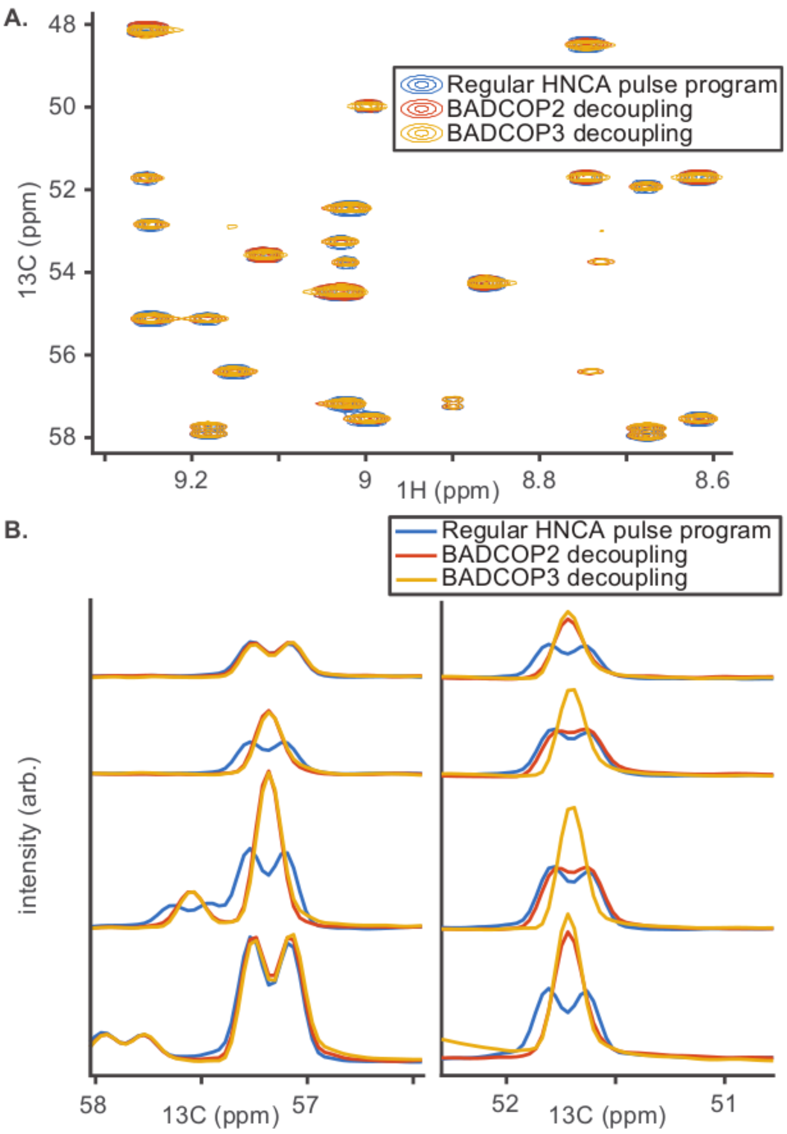

Supplementary Fig. 6. **Selective decoupling of C^β resolves degenerate correlations in the 2D H_N-C^α plane of HNCA for GB1.** (**A**) Three H_N-C^α planes with different C^β decoupling. Peaks change between singlets or doublets depending on their C^β chemical shift and the decoupling profile. (**B**) We found two cases of ambiguous sequential correlation for the standard HNCA (note that these would be dispersed in the 15N dimension for a full 3D HNCA, but genuine ambiguities still show up in 3D for more complicated systems). On the left, we see four overlapped peaks, i.e. the sequential correlations are ambiguous. Two peaks are reliably doublets irrespective of decoupling pulse (therefore they come from a serine or a theonine), and two other peaks are singlets for the same decoupling pulses (therefore they come from a system with $C^\beta \approx 30$ ppm). The correct sequential correlations must have the same behavior in all decoupling pulses. This resolves the ambiguity. On the right, we also see ambiguity for the regular HNCA which is resolved by comparing the line shapes under selective decoupling. Two of these peaks come from a system with $C^\beta \approx 30$ ppm, and the other two come from a system with $C^\beta \not\approx 30$ ppm.

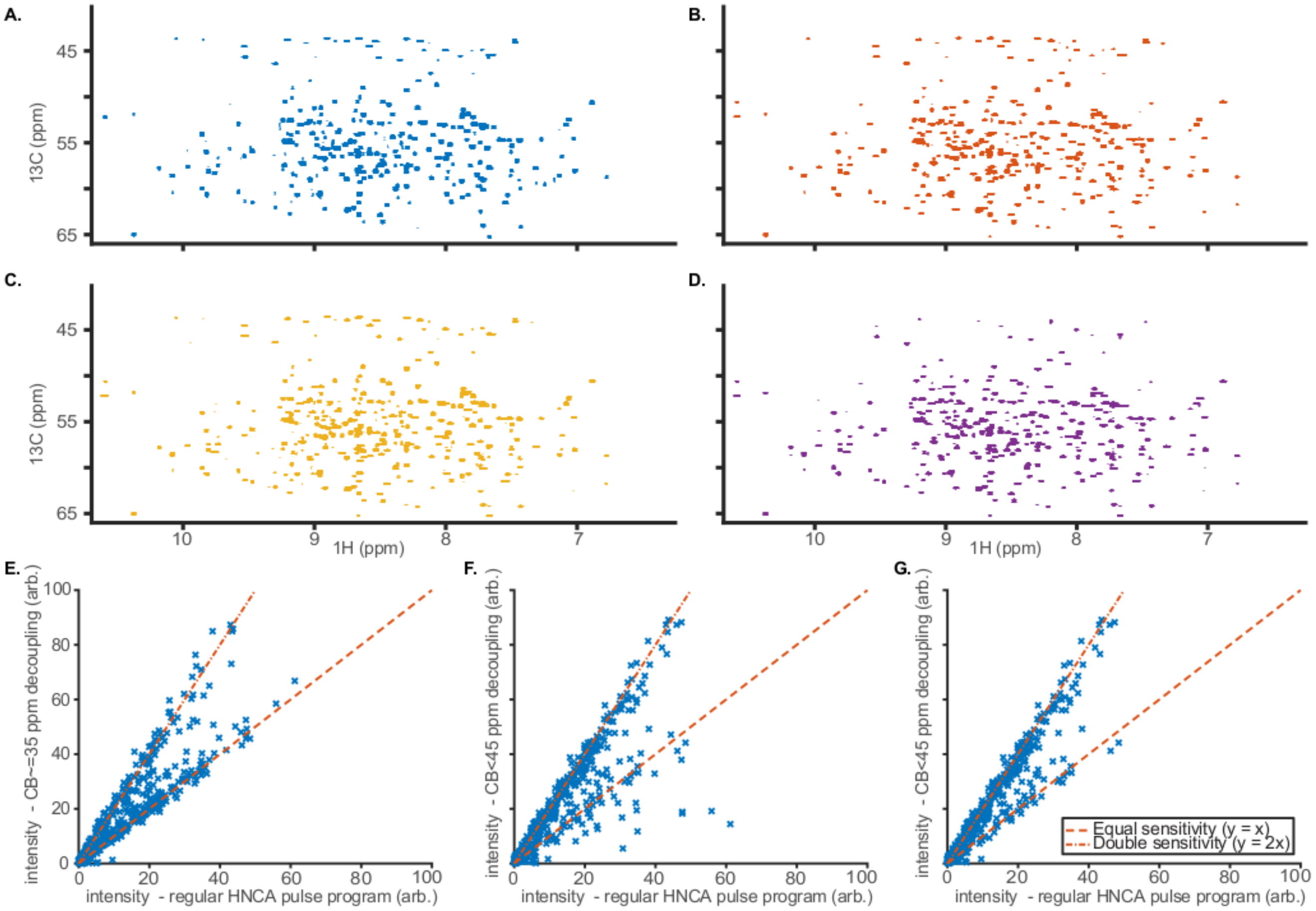

Supplementary Fig. 7. **Sensitivity of BADCOP1-3 versus standard implementation.** H_N-C^α planes of HNCA were acquired on a 700 μM sample of HCA, using (**A**) the standard Bruker pulse program as well as (**B**) with optimized decoupling of C^β below 35 ppm with BADCOP1, (**C**) with decoupling of $C^\beta \approx 30$ ppm with BADCOP2, and (**D**) with decoupling of C^β below 43 ppm with BADCOP3. (**E**) A comparison of the skyline projections of **A** and **C**, showing most peaks have similar sensitivity, but some have doubled sensitivity. (**F**) A comparison of the skyline projections of **A** and **D**, showing most peaks have doubled sensitivity, but some have reduced sensitivity, *below* the line $y = x$. These are the glycines. (**G**) The skyline projection was limited to 13C greater than 47 ppm, to exclude glycine. Now we see most peaks have doubled sensitivity using BADCOP3, while some have the same sensitivity as the regular pulse program. This is because serines and threonines are not decoupled. All acquisition, processing, and display settings are the same for the four spectra. Note that a comparison of **A** and **B** is given in Figure 1D in the main manuscript.

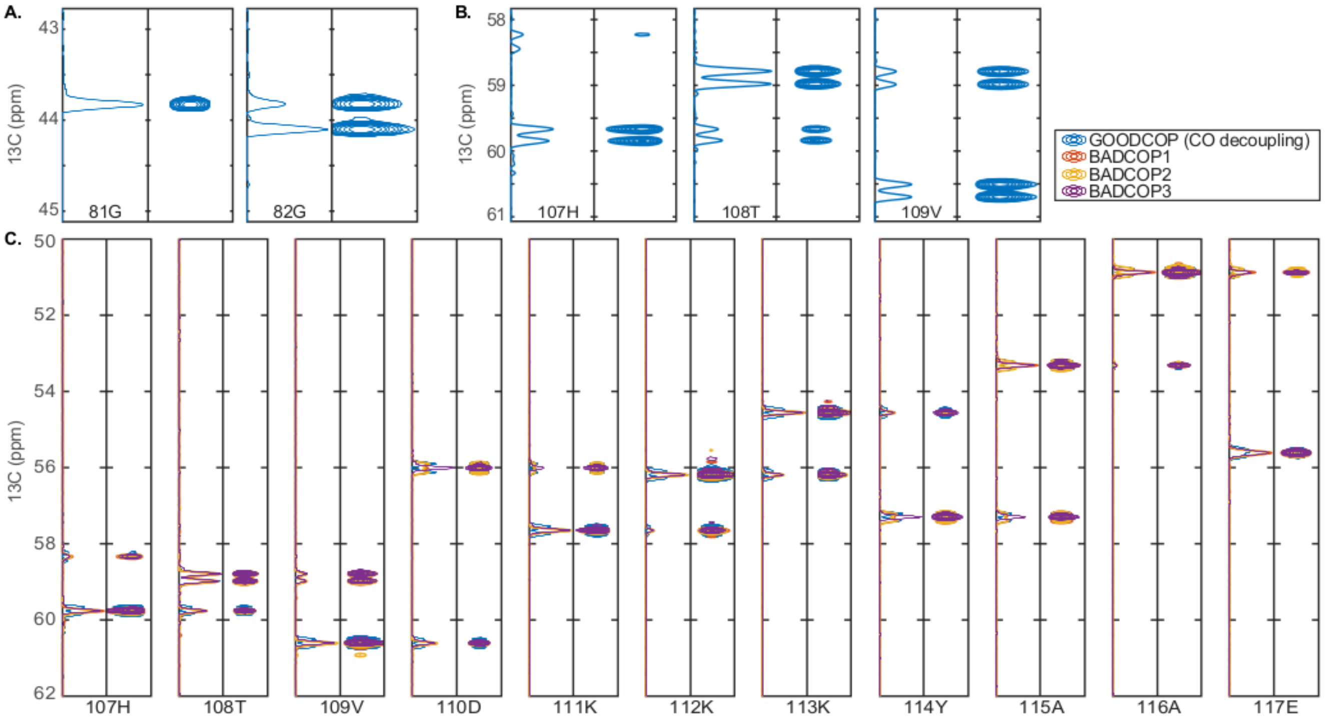

Supplementary Fig. 8. **Using BADCOP1-3 splitting patterns for backbone assignment.** Expanded version of Figure 5. Using line shape for assigning sequential matches to the primary sequence. (**A.**, **B.**) Same as in Figure 5 (**C.**) Using three BADCOP decoupling pulses gives residue-specific information. The three leftmost strips (the same strips as in **B**) show a particular sequence of different splitting patterns under the various decoupling pulses, as described in Figure 3A. This pattern is only consistent with one location on the primary sequence of HCAii, and so these can be unambiguously assigned. Further sequential matches show splitting patterns consistent with the primary sequence and the expected splitting patterns based on Figure 3A. Several other subsequences of splitting patterns visible in **C** are also unique in the primary sequence (e.g. 115A-116A; 113K-115A; 108T-112K). This gives many opportunities to make or confirm assignments, analogous to how the case of two sequential glycines was used in **A**. All acquisition and processing settings are the same for the four spectra, while strip contour levels are set individually for visual clarity.

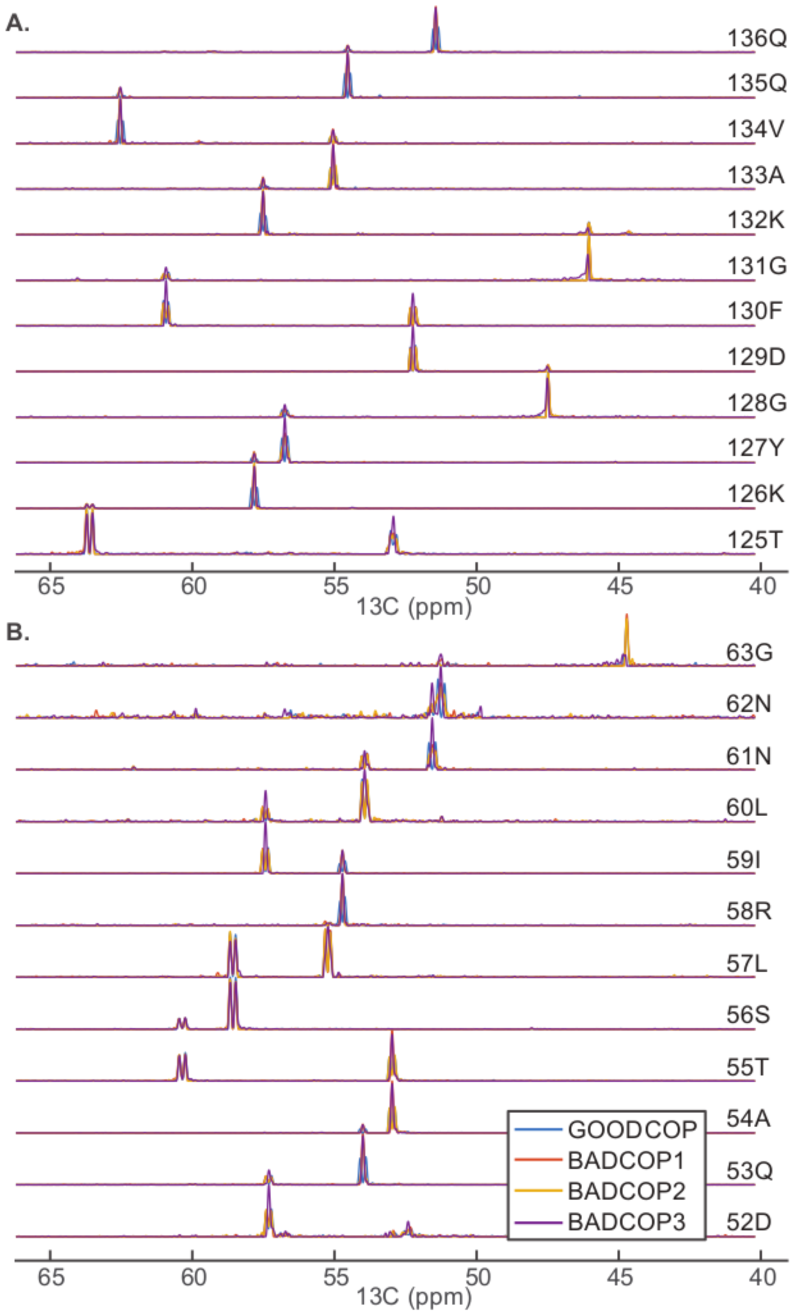

Supplementary Fig. 9. **Using BADCOP1-3 splitting patterns for backbone assignment.** (**A., B.**) Several examples of sequential assignments of HCAii, aided by residue specific line shape information. Distinctive patterns of doublets and singlets are produced by each decoupling pulse. Examining the chain of matches and their line shapes, it is straightforward to categorize individual strips based on the expected line shapes from Figure 3. Multiple short subsequences of splitting patterns visible here are unique in the primary sequence of HCAii (e.g. 131G-133A; 125T-128G; 54A-56S; 55T-57L; 57L-61N, and more). This allows multiple opportunities to make or confirm the assignments. Based on the HCAii primary sequence, all possible chains of six (or more) sequential correlations can be uniquely assigned based on line shapes produced by BADCOP1-3. However, most residues can be uniquely assigned so long as there are three or four sequential correlations in a row.

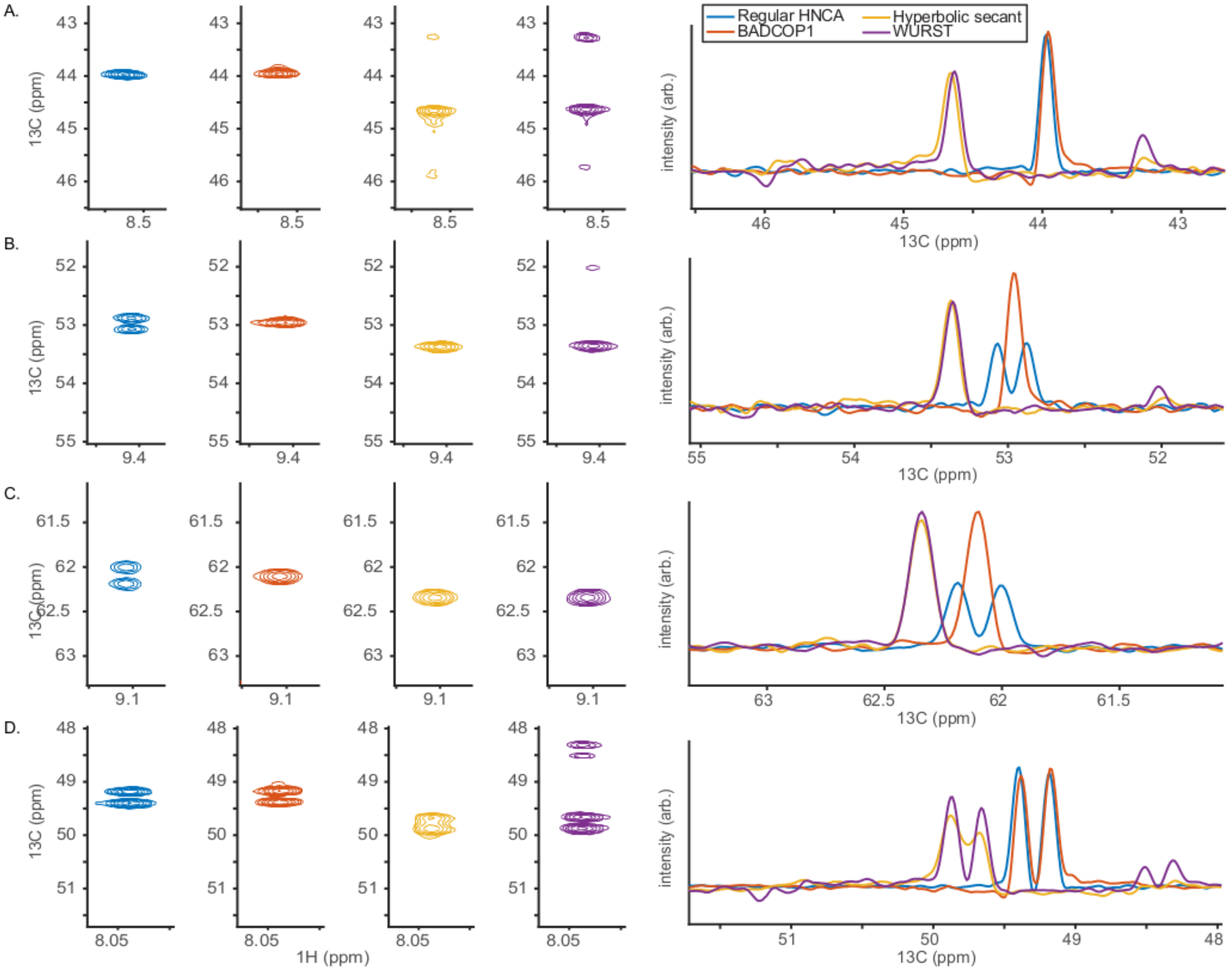

Supplementary Fig. 10. **Other selective decoupling pulses produce large Bloch-Siegert shifts.** Comparison of peak-positions of C^{β}-decoupled spectra using BADCOP1 versus WURST and hyperbolic secant decoupling. Each row (**A-D**) is the same peak, taken from four different spectra. The leftmost column used the standard Bruker pulse program, next is BADCOP1, then hyperbolic secant decoupling, and finally WURST decoupling. On the right, the same information is displayed as a 1D trace through the peak center, to better see the sensitivity and decoupling sidebands. (**A**) is a glycine peak. It appears unaffected by BADCOP1, but is shifted and reduced in height by both dynamical decoupling schemes. There are also quite large sideband peaks. (**B**) A non-glycine system that is collapsed into a singlet by BADCOP1, and also by the two dynamical decoupling schemes. However, only BADCOP1 keeps it in the correct position, doubles the sensitivity, and avoids sidebands. (**C**) A system in which all three decoupling methods have twofold sensitivity gain compared to the usual doublet, but only BADCOP1 avoids shifts. (**D**) Finally, we observe poor sensitivity and shifts even for non-decoupled peaks when we use dynamical decoupling (hyperbolic secant and WURST). However, BADCOP1 preserves the height and position, and does not generate sidebands. All acquisition, processing, and display settings are the same for the four spectra.

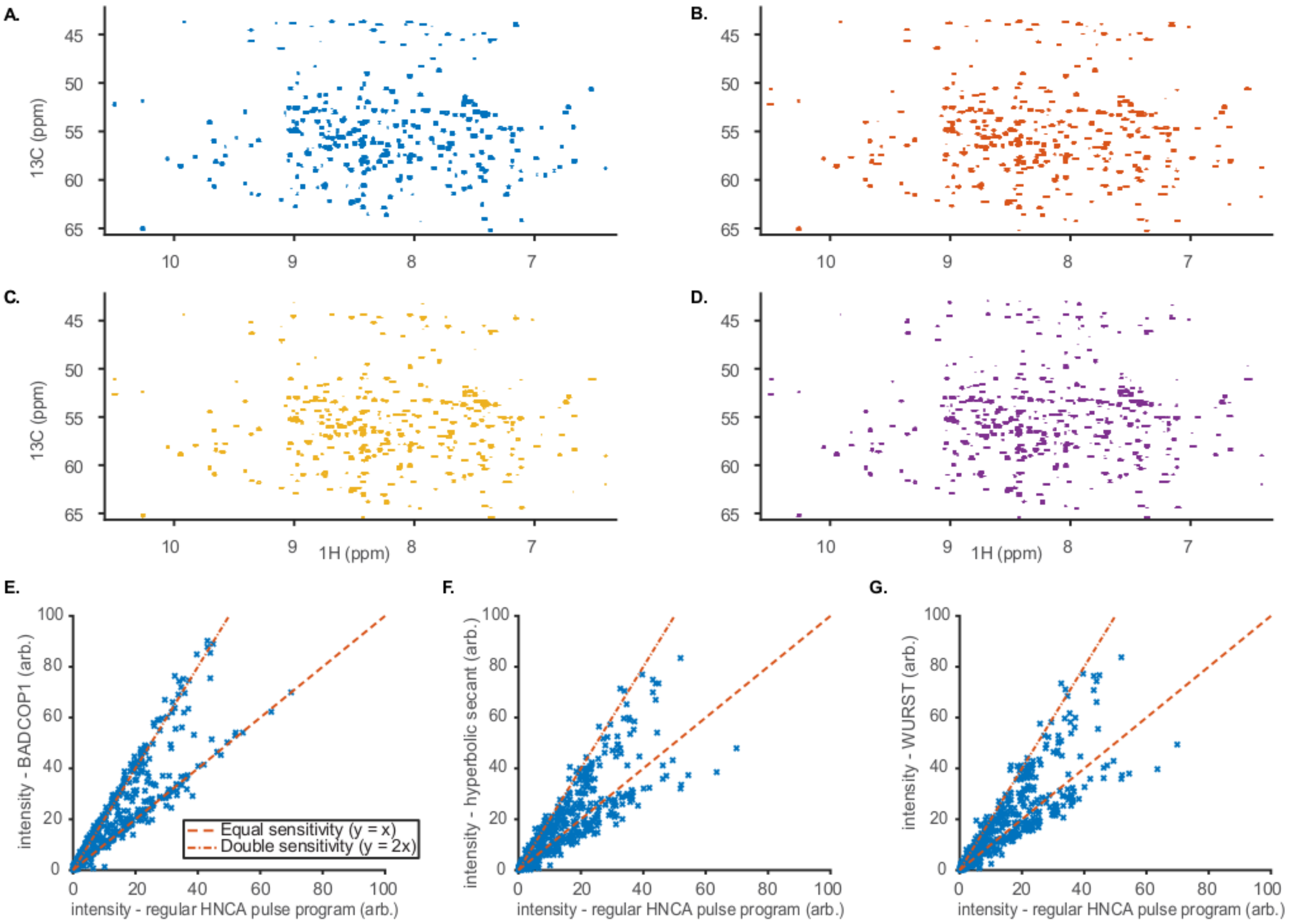

Supplementary Fig. 11. **BADCOP has better sensitivity than other selective decoupling pulses.** Comparison of sensitivity of C^{β}-decoupled spectra using BADCOP1 versus WURST and hyperbolic secant decoupling. (**A**) A 2D plane (first 15N increment) of the HNCA of protein HCAii, acquired using the standard Bruker pulse program. (**B**) The same, but acquired using BADCOP1. (**C**) The same, but acquired using a two-lobe hyperbolic secant decoupling of 30 ppm width (per lobe), centered on 170 and 20 ppm. (**D**) The same, but acquired using a two-lobe WURST decoupling with the same lobes. (**E-G**) Comparisons of the skyline projections of each decoupling scheme versus the standard pulse program. Only for BADCOP1, **E**, do the two broad trends follow the lines of equal and double sensitivity. The two dynamical decoupling pulses apparently exhibit generally lower sensitivity than BADCOP1, i.e. the two trends fall below the lines of equal and double sensitivity. For the other decoupling pulses, **F**, **G**, we see significant numbers of points *below* the line $y = x$, indicating that decoupling has actually reduced sensitivity for many peaks. All acquisition, processing, and display settings are the same for the four spectra.
